# Interferon-γ Restricts *Toxoplasma gondii* Development in Murine Skeletal Muscle Cells via Nitric Oxide Production and Immunity-Related GTPases

**DOI:** 10.1371/journal.pone.0045440

**Published:** 2012-09-14

**Authors:** Anna C. Takács, Izabela J. Swierzy, Carsten G. K. Lüder

**Affiliations:** Institute for Medical Microbiology, University Medical Center, Georg-August-University, Göttingen, Germany; University of Oklahoma Health Sciences Center, United States of America

## Abstract

The apicomplexan parasite *Toxoplasma gondii* is regularly transmitted to humans via the ingestion of contaminated meat products from chronically infected livestock. This route of transmission requires intracellular development and long-term survival of the parasite within muscle tissue. In this study, we determined the cell-autonomous immunity of mature primary embryonic or C2C12 skeletal muscle cells (SkMCs) to infection with *T. gondii*. Non-activated SkMCs and control fibroblasts sustained parasite replication; however, interferon (IFN)-γ significantly inhibited parasite growth in SkMCs but not in fibroblasts. Intracellular parasite replication was diminished by IFN-γ whereas host cell invasion was not affected. Tumor necrosis factor (TNF) did not further increase the IFN-γ-triggered host defense of SkMCs against *Toxoplasma*. Remarkably, IFN-γ alone or in combination with TNF decreased the high level of *T. gondii* bradyzoite formation being observed in non-activated SkMCs. Stimulation of SkMCs with IFN-γ strongly triggered expression of inducible nitric oxide synthase (iNOS) transcripts, and induced significantly higher levels of nitric oxide (NO) in SkMCs than in fibroblasts. Consequently, pharmacological inhibition of iNOS partially abrogated the IFN-γ-induced toxoplasmacidal activity of SkMCs. In addition, SkMCs strongly up-regulated immunity-regulated GTPases (IRGs) following stimulation with IFN-γ. IRGs accumulated on *Toxoplasma*-containing vacuoles in SkMCs in a parasite strain-dependent manner. Subsequent vacuole disruption and signs of degenerating parasites were regularly recognized in IFN-γ-treated SkMCs infected with type II parasites. Together, murine SkMCs exert potent toxoplasmacidal activity after stimulation with IFN-γ and have to be considered active participants in the local immune response against *Toxoplasma* in skeletal muscle.

## Introduction

Skeletal muscle plays a critical role in the transmission of the zoonotic parasite *Toxoplasma gondii* to humans. Between 30% and 63% of human infections have been related to the consumption of undercooked or cured meat products as revealed by a multi-centre study involving acutely infected pregnant women and non-infected controls [1]. Although infection is mostly asymptomatic or benign, the parasite is a significant threat for individuals with a premature or suppressed immune system and can lead to severe and life-threatening toxoplasmosis. Transmission of *T. gondii* to humans via the ingestion of contaminated meat products may depend on the development and long-term survival of parasites in skeletal muscle cells (SkMCs) of chronically infected livestock and poultry. We have shown previously that these cells, after differentiation *in vitro* to mature myotubes, indeed provide a niche which sustains intracellular development and differentiation to the bradyzoite stage of the parasite [2].

During embryogenesis or following muscle injury, SkMCs transform from proliferating and fusogenic stem cells, i.e. myoblasts to multinucleated myotubes which further differentiate to large syncytial muscle fibers [3]. Mature SkMCs provide a unique immunological environment for the development of pathogens, with no detectable expression of major histocompatibility complex (MHC) class I and class II expression under physiological conditions [4]. Furthermore, expression of HLA-G or the B7 homologue B7-H1 (PD-L1) by human myoblasts fulfils tolerizing or even suppressive functions within muscle tissue [5], [6]. Limited immune reactions in skeletal muscle may thus facilitate long-term survival of *Toxoplasma* and make this organ to one of the preferred body sites where tissue cysts persist until orally ingested by a new host [7]. Under certain conditions, i.e. after activation by proinflammatory cytokines *in vitro* or during inflammatory myopathies *in vivo*, however, muscle cells can express MHC class I and II antigens and present antigens to T cells [8], [9]. In addition, they express a variety of cell surface receptors and soluble immune mediators and thus fulfil requirements for immunocompetence. During reactivation of chronic toxoplasmosis or during acute disease following recent infection, the occurrence of polymyositis has been well established in both humans and animals [10], [11], [12], [13], [14]. Although immune cells infiltrating the infected muscle tissue certainly mediate much of the local inflammatory response, this supports the view that SkMCs contribute to the immune response to *T. gondii* within muscle tissue and may be pivotal during toxoplasmic myopathies. However, the impact of SkMCs in the local host response to *Toxoplasma* and host factors or molecular mechanisms which might limit parasite development in SkMCs have not yet been determined.

Resistance to infection with obligate intracellular *Toxoplasma* parasites largely depends on Th1-type cell-mediated immune responses. Interferon (IFN)-γ released from CD4+ and CD8+ T lymphocytes is the most critical mediator of immunity against *T. gondii* [15], [16], [17], [18]. Early during infection, natural killer (NK) cells are the main producers of IFN-γ and are important mediators of innate immunity [19]. IFN-γ activates effector cells of both hematopoietic and non-hematopoietic origin to exert anti-*Toxoplasma* activity [20]. Tumor necrosis factor (TNF), interleukin (IL)-1 and IL-6 synergize with IFN-γ to strengthen the anti-parasitic response [21], [22]. They exert anti-parasitic activity by up-regulating the expression of effector molecules in various cell types. Depending on the host species, control of intracellular *T. gondii* is mediated by production of nitric oxide (NO) by the inducible NO synthase (iNOS) [23], [24], disruption of the parasitophorous vacuole by immunity-related GTPases (IRGs; formerly called p47 GTPases) and p65 guanylate-binding proteins (GBPs; also called p65 GTPases) [25], [26], [27], tryptophan starvation via up-regulation of the indoleamine 2,3-dioxygenase (IDO) [28], production of oxygen radicals [29], and activity of P2X_7_ receptors [30].

In this study, we determined the impact of IFN-γ and TNF on the development of *T. gondii* in mouse SkMCs that have been differentiated *in vitro* to mature myotubes. The results show that IFN-γ readily activates muscle cells to restrict parasite replication but does not trigger differentiation from the rapidly replicating tachyzoite to the slowly replicating bradyzoite stage. NO production mediated by iNOS and disruption of the *Toxoplasma* PV by IRG activity may be instrumental in restricting parasite propagation in SkMCs. These results establish SkMCs as immunocompetent effector cells in the response to *Toxoplasma* within skeletal muscle.

## Results

### In vitro differentiation of SkMCs

**Figure 1:**
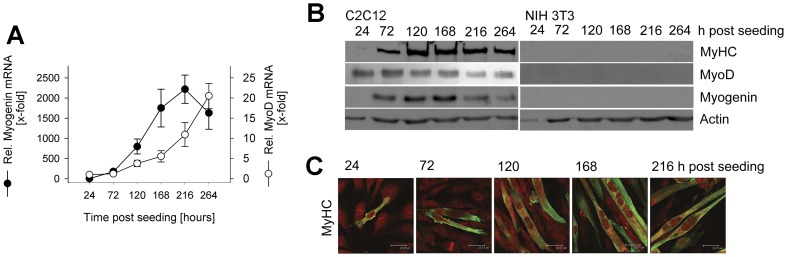
In vitro differentiation of mouse-derived C2C12 SkMCs. C2C12 myoblasts were cultivated in differentiation medium from 24 hours after seeding onwards for different periods of time as indicated. NIH/3T3 fibroblasts were cultivated in parallel and were used as controls. (A) Total RNA was isolated, and after reverse transcription of mRNA, cDNA was amplified by quantitative real-time PCR. The relative abundance of myogenin (filled symbols) and MyoD (open symbols) mRNA was normalized to that of β-actin mRNA, and was compared to the mRNA level before differentiation, i.e. at 24 hours post seeding. Data represents means ± S.E.M. from three independent experiments. (B) Complete cellular protein extracts were separated by SDS-PAGE, transferred to nitrocellulose, and labeled with antibodies recognizing muscle cell-specific myosin heavy chain (MyHC), MyoD, myogenin, or β-actin as loading control. Immune complexes were visualized with HRPO-conjugated secondary antibodies and enhanced chemiluminescence. Results from a representative experiment out of three are depicted. (C) After fixation, cells were immunolabeled using anti-MyHC primary antibody and Cy2-conjugated secondary antibody (green fluorescence), and were counterstained using propidium iodide (red fluorescence). Representative images were obtained by confocal laser scanning microscopy.

Differentiation of primary embryonic skeletal muscle cells after *in vitro* cultivation for 6 days has been shown previously by the presence of multinucleated myotubes and the up-regulation of muscle-specific transcription factors MyoD and Myf5 [2]. Here, we also determined the differentiation of C2C12 mouse myoblasts to mature myotubes. The results show that transfer of C2C12 myoblasts into differentiation medium induced significant levels of myogenin mRNA 72 hours after seeding which further increased during the following 6 days (Fig. 1A). Up-regulation of mRNA of the basic helix-loop-helix transcription factor MyoD was slightly delayed as compared to myogenin mRNA but also continuously increased starting from 120 hours post seeding until the end of the observation (Fig. 1A). Immunoblotting confirmed the expression of muscle-specific transcription factors myogenin and MyoD during cultivation of C2C12 cells in differentiation medium, with the highest levels being observed between 72 and 168 hours after seeding the cells (Fig. 1B). In addition, myosin heavy chain (MyHC) which is indicative for terminal differentiation of SkMCs was expressed from 72 hours after seeding onwards with highest levels being observed from 120 hours onwards (Fig. 1B). Immunofluorescence microscopy confirmed expression of MyHC in an increasing number of cells (Fig. 1C). Most of these cells were highly elongated and contained multiple nuclei indicating formation of mature myotubes. NIH/3T3 control fibroblasts did not express any of the muscle-specific proteins MyoD, Myf5 or MyHC as expected (Fig. 1B). Together, the data shows that embryonic progenitors and C2C12 myoblasts can differentiate *in vitro* to mature myotubes and represent valuable models to investigate the interaction of *T. gondii* with SkMCs.

### IFN-γ restricts T. gondii replication in SkMCs

**Figure 2:**
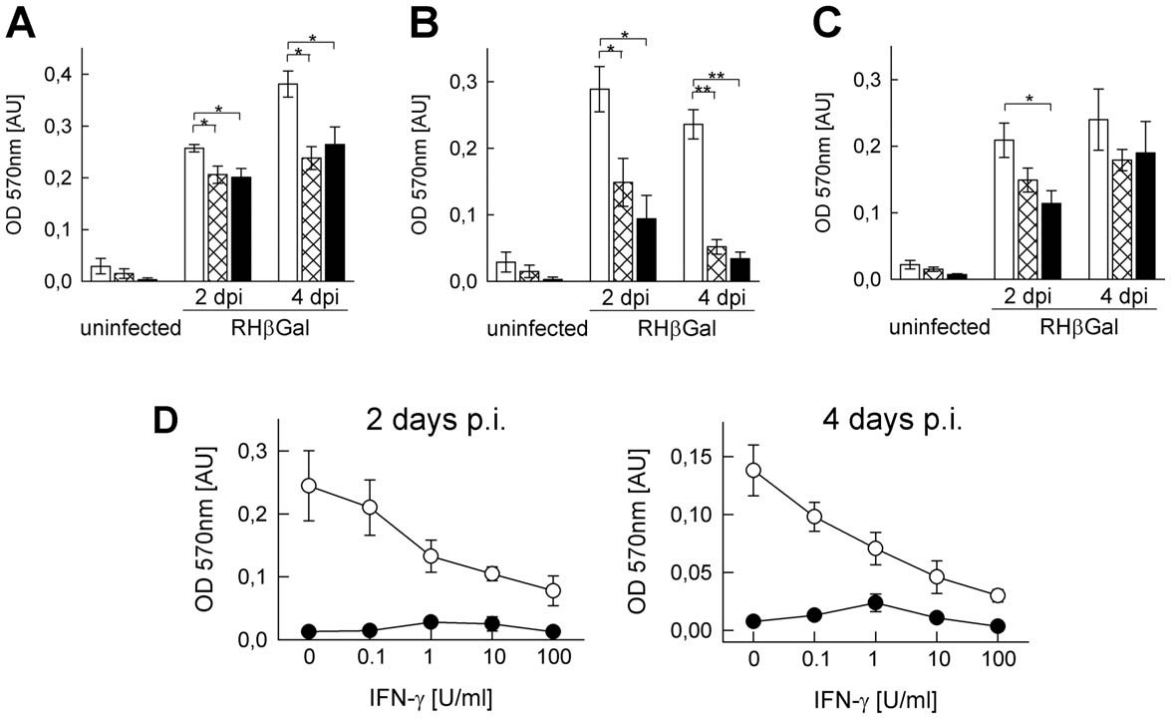
IFN-γ restricts *T. gondii* development in differentiated SkMCs more vigorously than in fibroblasts. *In vitro* differentiated primary embryonic SkMCs (A) or C2C12 SkMCs (B, D) and control NIH/3T3 fibroblasts (C) were infected or not with transgenic RH parasites expressing β-galactosidase (RHβGal; parasite-host cell ratio 2∶1). (A–C) At the time of infection, cells were stimulated with 100 U/ml IFN-γ alone (cross-hatched bars) or along with 100 pg/ml TNF (black bars), or were left non-stimulated (open bars). At 2 or 4 days post infection (dpi), growth of β-galactosidase-expressing *T. gondii* was quantitated by a colorimetric CPRG assay. Bars represent means ± S.E.M. from at least three independent experiments. Significant differences between non-stimulated and stimulated cells are indicated (*p<0.05; **p<0.01). (D) Infected C2C12 SkMCs (open symbols) or non-infected controls (closed symbols) were stimulated with 0–100 U/ml IFN-γ for 2 or 4 days. Parasites were quantitated as described above. Results are means ± S.E.M. from 5 independent experiments.

**Figure 3:**
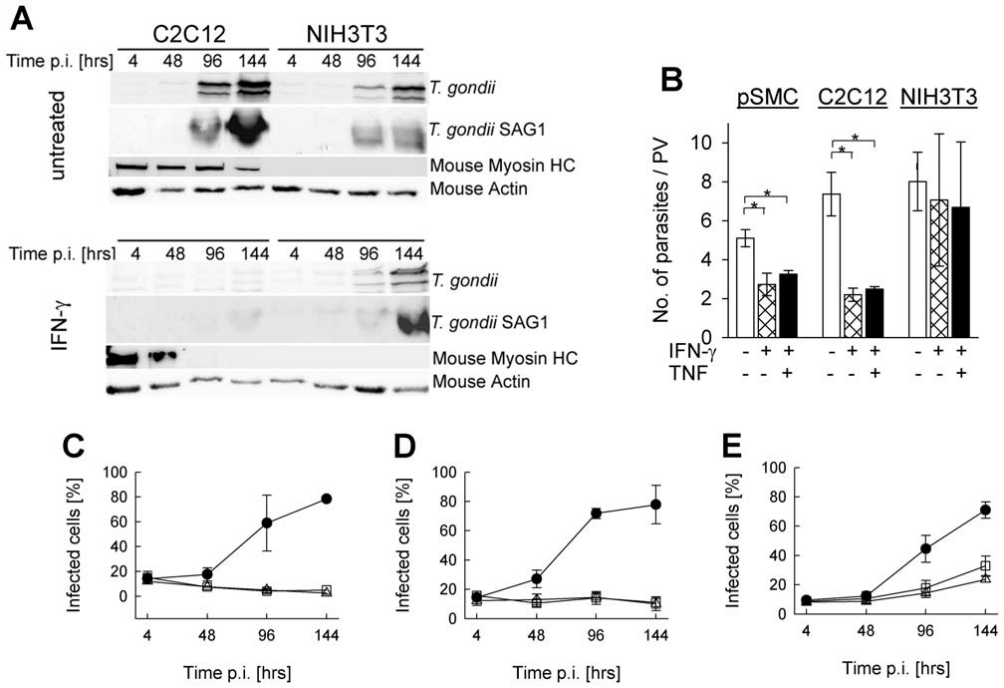
Impact of IFN-γ and TNF on *Toxoplasma* development in SkMCs. Primary embryonic SkMCs or C2C12 SkMCs were differentiated to mature myotubes. They were, together with control NIH/3T3 fibroblasts, infected with *T. gondii* NTE tachyzoites (parasite-host cell ratio 2∶1). At the time of infection, cells were stimulated or not with 100 U/ml IFN-γ alone or combined with 100 pg/ml TNF. (A) At different time points post infection (p.i.), C2C12 and NIH/3T3 cells were lysed, and soluble proteins were separated by SDS-PAGE and transferred to nitrocellulose membranes. Membranes were probed with anti-*T. gondii* serum, anti-*T. gondii* SAG1, anti-mouse myosin HC, or anti-mouse actin and appropriate HRPO-conjugated secondary antibodies. Immune complexes were visualized by enhanced chemiluminescence. (B) Forty-eight hours after infection, cells were fixed and permeabilized, and *T. gondii* was visualized by immunofluorescence staining. The total cell population was stained with propidium iodide. The number of parasites was counted in at least 100 parasitophorous vacuoles (PV) per sample. Data represents the mean number of parasites per PV ± S.E.M. from at least 3 independent experiments; significant differences between non-stimulated and stimulated cells are indicated (*p<0.05). (C–E) At the time of infection, primary SkMCs (C), C2C12 (D) or NIH/3T3 (E) cells were stimulated with IFN-γ alone (open squares), IFN-γ combined with TNF (open triangles), or were left untreated (closed symbols). Cells were fixed at different time points after infection, and *T. gondii* and total cells were visualized by immunolabelling and propidium iodide staining, respectively. After inspection of at least 500 cells per sample, the percentages of infected cells were calculated. Results are means ± S.E.M. from at least 3 independent experiments.

In order to unravel the impact of IFN-γ and TNF on *Toxoplasma* development within muscular tissue, we determined parasite propagation in *in vitro* differentiated SkMCs and control fibroblasts. Since mouse-virulent type I *T. gondii* strains are able to resist distinct defense mechanisms [31], [32], we first used *lacZ*-transgenic parasites on the type I RH background. This enabled us to determine whether SkMCs are able to even restrict replication of relatively resistant parasite strains. Following treatment of primary mouse-derived myotubes with 100 U/ml IFN-γ, β-galactosidase reporter activity at 2 and 4 days post infection was significantly reduced as compared to untreated controls (p<0.05; Students *t*-test; Fig. 2A). IFN-γ-mediated inhibition of parasite propagation was even more pronounced in differentiated C2C12 cells leading to β-galactosidase activity of only ∼52% (*p*<0.05) and ∼22% (*p*<0.01) of untreated controls at 2 or 4 days p.i., respectively (Fig. 2B). Although IFN-γ also restricted parasite growth in control fibroblasts, this reduction was statistically not significant (Fig. 2C). Dose-response analyses of the effect of IFN-γ at 2 and 4 days of treatment indicated an increasing anti-parasitic activity of the cytokine from as little as 0.1 U/ml to 100 U/ml, with the highest concentration largely abrogating parasite propagation (Fig. 2D). TNF enhanced IFN-γ activity only to a minor extent in C2C12 SkMCs and NIH/3T3 fibroblasts, but had no synergistic effect in primary SkMCs (Fig. 2A–C) suggesting that TNF is not of major relevance for limiting parasite propagation in SkMCs. Further experiments using wild type NTE parasites confirmed the ability of IFN-γ-activated SkMCs to restrict *Toxoplasma* replication and extended the above findings to mouse-avirulent type II strains. Since these parasites have a longer duplication time as compared to type I parasites, their use also enabled us to extend the observation periods without extensive host cell lysis. Immunoblot analyses confirmed a strong anti-*Toxoplasma* activity of IFN-γ in SkMCs. The abundance of soluble *Toxoplasma* proteins including the predominant surface antigen SAG1 indeed strongly increased in non-activated C2C12 myotubes, but not in those concomitantly activated with IFN-γ (Fig. 3A). In contrast, the levels of parasite proteins in infected NIH/3T3 similarly increased in IFN-γ-treated NIH/3T3 as in untreated controls. We also recognized that following treatment of differentiated SkMCs with IFN-γ, expression of myosin HC decreased considerably. Together, the data established that IFN-γ fulfils anti-parasitic activity against *T. gondii* in SkMCs and also influences the expression of muscle-specific marker proteins.

Propagation of *T. gondii* in IFN-γ-stimulated SkMCs can be restricted by decreased invasion or decreased intracellular replication. In order to distinguish between these possibilities, we fluorescently labeled *T. gondii* (strain NTE) following infection of SkMCs or fibroblasts for different periods of time and analyzed them microscopically. The results show significantly lower average numbers of parasites per parasitophorous vacuole in IFN-γ-treated primary SkMCs or C2C12 cells as compared to the untreated controls (*p*<0.05, Student's *t*-test; Fig. 3B). In contrast, the number of parasites per vacuole was only slightly reduced by IFN-γ in NIH/3T3 cells at 2 days post infection (*p*>0.05). TNF did not further reduce IFN-γ-regulated *T. gondii* replication in SkMCs or fibroblasts (Fig. 3B).

As reported previously, *T. gondii* efficiently invaded non-activated SkMCs [2], and this was similarly observed at 4 hours post infection in IFN-γ-activated SkMCs (Fig. 3C–D). From 48 hours onwards, the percentages of infected primary SkMCs and C2C12 cells strongly increased in the absence of IFN-γ, which reflected considerable parasite replication (see above), subsequent host cell lysis and infection of new host cells (Fig. 3C–D). In sharp contrast, following activation with IFN-γ alone or in combination with TNF, the percentages of infected cells slightly decreased. Furthermore, the percentages of infected cells increased in both non-activated and activated fibroblasts although the increase was clearly lower after activation with IFN-γ or IFN-γ plus TNF as compared to non-activated controls (Fig. 3E). These results collectively established a major impact of IFN-γ but not TNF on the parasite replication in differentiated SkMCs.

### Bradyzoite formation is not accelerated in activated SkMCs

**Figure 4:**
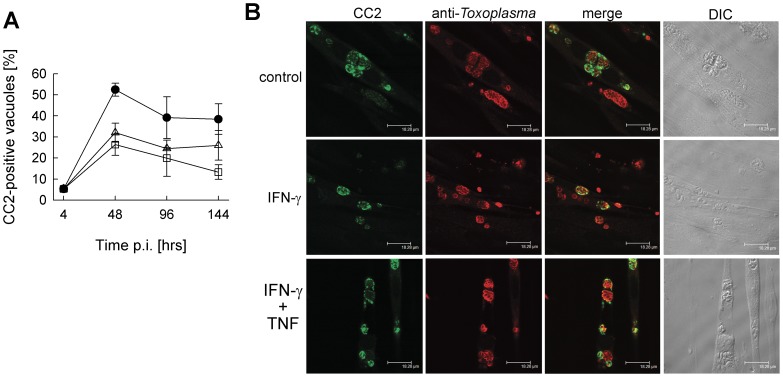
*Toxoplasma* bradyzoite formation in SkMCs after stimulation with IFN-γ and TNF. Differentiated C2C12 SkMCs were infected with *T. gondii* NTE tachyzoites, and were stimulated with 100 U/ml IFN-γ alone (open squares), or in combination with TNF (open triangles), or were left non-stimulated (closed circles). At different time points after infection, cells were fixed and permeabilized, and *T. gondii* stage conversion was assessed by immunolabelling bradyzoite-containing vacuoles with CC2 monoclonal antibody (green fluorescence) and the total parasite population with polyclonal anti-*Toxoplasma* serum (red fluorescence). (A) The percentage of bradyzoite-containing vacuoles was calculated after inspection of at least 100 parasitophorous vacuoles per sample. Data represents means ± S.E.M. from four independent experiments. (B) Representative images of *T. gondii* vacuoles from each condition at 48 hours post infection are shown.

In distinct cell types, proinflammatory cytokines including IFN-γ and TNF are appropriate triggers to induce stage differentiation from fast-replicating tachyzoites to dormant bradyzoites. We therefore wondered whether reduced replication of *T. gondii* in IFN-γ-treated SkMCs is accompanied by conversion towards the bradyzoite stage. For these experiments we used the NTE strain of *T. gondii*, since type II strains convert more readily to bradyzoites than type I strains. Double immunofluorescence microscopy using stage-specific antibodies showed that differentiated C2C12 SkMCs cultivated in the absence of any exogenous stimulus triggered high levels of bradyzoite formation already at 2 days after infection (Fig. 4A), thus confirming previous findings [2]. Differentiation towards the bradyzoite stage was most often observed in vacuoles which harbored 2 to 8 parasites after two days of infection, whereas larger vacuoles and single parasites mostly did not react with the CC2 antibody (Fig. 4B, upper panel). Remarkably, the percentage of bradyzoite-containing vacuoles was considerably lower when SkMCs had been previously activated with IFN-γ or IFN-γ along with TNF (Fig. 4A). This was likely due to an increased appearance of vacuoles containing only one parasite (Fig. 4B) the latter being unable to convert to the bradyzoite stage [33], [34]. Lower levels of bradyzoite-containing vacuoles after IFN-γ or IFN-γ/TNF activation of SkMCs as compared to non-activated SkMCs persisted until 144 hours after infection. We also recognized a general reduction in the number of bradyzoite-containing vacuoles during extended infection times and this was partially related to parasite-mediated host cell lysis and infection of new SkMCs. In conclusion, IFN-γ alone or combined with TNF appears inappropriate to enhance *T. gondii* bradyzoite formation in differentiated SkMCs.

### IFN-γ-triggered iNOS activity restricts T. gondii development in SkMCs

**Figure 5:**
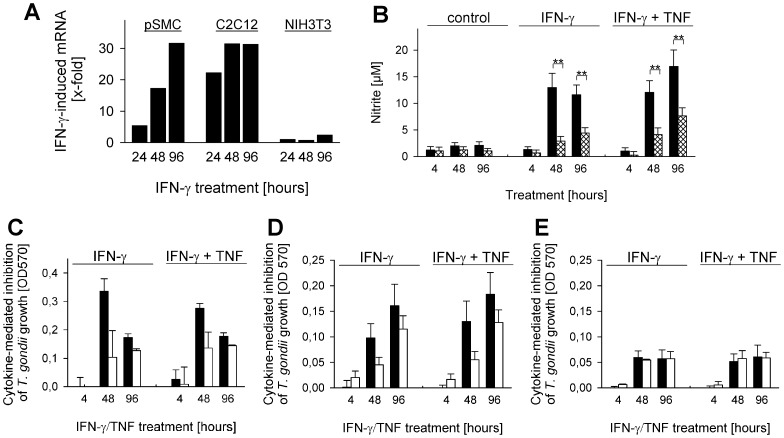
IFN-γ-triggered expression and activity of iNOS partially restricts *Toxoplasma* propagation in SkMCs. Primary embryonic SkMCs (pSMC) or C2C12 SkMCs were differentiated to mature myotubes. Differentiated SkMCs and control NIH/3T3 fibroblasts were infected at a parasite-host cell ratio of 2∶1 with transgenic *T. gondii* RH tachyzoites expressing β-galactosidase as a reporter (C–E), or were left non-infected (A,B). Cells were stimulated or not with 100 U/ml IFN-γ alone or combined with 100 pg/ml TNF. (A) Total RNA was isolated at the indicated time points, mRNA was reverse transcribed, and iNOS or β-actin cDNA was amplified by quantitative real-time PCR. Data represents the mean induction of iNOS mRNA by IFN-γ normalized to β-actin; data is from a representative experiment out of two. (B) Nitrite was measured by the Griess reaction in culture supernatants obtained from non-infected C2C12 (black bars) and non-infected NIH/3T3 cells (cross-hatched bars) at the indicated time points. Data represents means ± S.E.M. from at least 5 independent experiments; significant differences between C2C12 and NIH/3T3 cells are indicated (**p<0.01). (C–E) Primary SkMCs (C), C2C12 (D) and NIH/3T3 cells (E) were treated with l-NIL (open bars), or were left untreated (black bars) at 1 hour prior to infection and cytokine stimulation. At different time points after infection/cytokine stimulation, growth of β-galactosidase-expressing *T. gondii* was quantitated by a colorimetric CPRG assay. The inhibition of parasite growth triggered by IFN-γ alone or in combination with TNF was calculated by subtracting OD570 values of cytokine-stimulated cultures from that of non-stimulated control cultures; bars represent means ± S.E.M. from at least two independent experiments.

Depending on the host species and the cell type, several effector mechanisms including the production of reactive nitrogen intermediates contribute to the anti-parasitic activity of IFN-γ in *Toxoplasma*-infected cells [24], [35]. We therefore examined whether expression and activity of iNOS, the IFN-γ-regulated isoform of nitric oxide synthases might also contribute restricting *T. gondii* propagation in differentiated murine SkMCs. Stimulation of primary embryonic SkMCs or C2C12 SkMCs with IFN-γ led to much higher levels of iNOS mRNA at 24, 48 and 96 hours as compared to fibroblasts (Fig. 5A). Consequently, nitric oxide (NO) was produced by IFN-γ-activated C2C12 SkMCs at significantly higher concentrations than by IFN-γ-treated fibroblasts as revealed by measuring nitrite, a stable end product of NO (Fig. 5B; *p*<0.01). Additional stimulation with TNF increased IFN-γ-triggered NO production in both SkMCs and fibroblasts slightly, and NO levels were again higher in SkMCs than in fibroblasts (*p*<0.01). In contrast, only low levels of NO were produced by non-activated SkMCs and fibroblasts, and these did not differ significantly between both cell types.

In order to unravel the functional importance of IFN-γ-triggered NO production in restricting *Toxoplasma* development in SkMCs, iNOS activity was inhibited using l-NIL. Control experiments indicated that 100 µM l-NIL indeed completely abrogated IFN-γ-induced NO production in SkMCs and fibroblasts (data not shown). Infection assays were performed with β-galactosidase-expressing RH parasites since their propagation can be readily quantified, and since type I and type II *Toxoplasma* strains are both susceptible to iNOS-mediated NO production [24], [35], [36]. Infection of primary and C2C12 permanent SkMCs with these parasites in the absence of l-NIL confirmed that IFN-γ alone or in combination with TNF inhibited parasite propagation at 2 and 4 days post infection (Fig. 5C,D). Importantly, prior treatment of SkMCs with l-NIL however decreased the IFN-γ- or IFN-γ/TNF-mediated restriction of parasite replication considerably. In contrast, cytokine stimulation of fibroblasts inhibited parasite propagation only to a minor extent, and this was not altered after treating cells with l-NIL (Fig. 5E). Together, these results establish a role for iNOS activity in restricting *Toxoplasma* development in IFN-γ-activated SkMCs.

### Accumulation of IRG proteins on Toxoplasma PVs in IFN-γ-activated SkMCs

**Figure 6:**
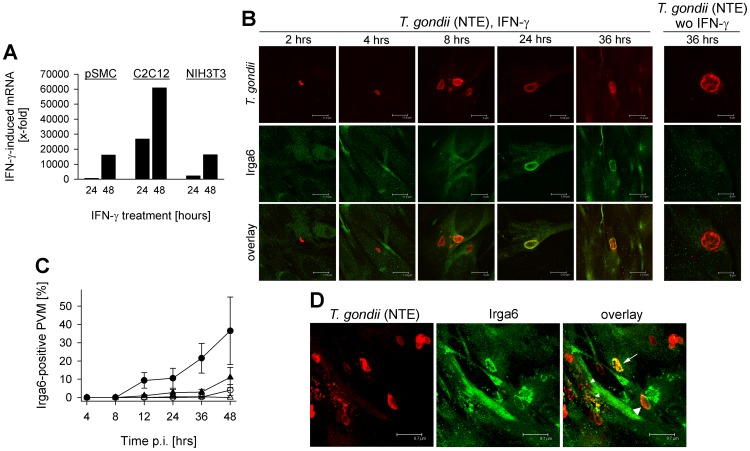
Expression and activity of p47 GTPases contribute to IFN-γ-mediated growth inhibition of *Toxoplasma* type II parasites in SkMCs. Primary embryonic SkMCs (pSMC) or C2C12 SkMCs were differentiated to mature myotubes, and were infected at a parasite-host cell ratio of 2∶1 with *T. gondii* NTE parasites (B–D), or were left non-infected (A). NIH/3T3 fibroblasts were used as non-SkMC controls. At the time of infection, cells were stimulated or not with 100 U/ml IFN-γ. (A) After isolation of total RNA, mRNA was reverse transcribed, and Irga6 or β-actin cDNA was amplified by quantitative real-time PCR. Data represents the mean induction of Irga6 mRNA by IFN-γ normalized to β-actin; data is from a representative experiment out of two. (B) At different time points after infection, C2C12 cells were fixed, and Irga6 (green fluorescence) and *T. gondii* (red fluorescence) were immunolabelled. Representative images of both labels were simultaneously obtained by confocal laser scanning microscopy and were then superimposed (overlay). (C) Percentages of Irga6-positive parasitophorous vacuolar membranes (PVM) were determined microscopically as described above by examining at least 100 vacuoles per sample in C2C12 SkMCs (circles) or NIH/3T3 fibroblasts (triangles), either stimulated with 100 U/ml IFN-γ (closed symbols) or left non-stimulated (open symbols). Data represents means ± S.E.M. from at least three independent experiments. (D) *Toxoplasma*-infected C2C12 SkMCs were stimulated with 100 U/ml IFN-γ, were fixed at 36 hours post infection, and were stained as described above. Images of different focal planes were recorded by confocal laser scanning microscopy and were superimposed using a maximum projection algorithm. Irga6-positive parasitophorous vacuoles (NTE parasites) that show no signs of disruption or that appear disrupted are indicated by large arrow heads and arrows, respectively. Putatively disintegrated parasites are also indicated (small arrow heads).

IFN-γ-mediated expression and subsequent translocation of IRG proteins (p47 GTPases) to parasite-containing vacuoles have been recently recognized as an important defense mechanism of mice against mouse-avirulent *Toxoplasma* strains [25], [26], [37]. In order to unravel the significance of this mechanism in SkMCs, we first determined cell type-specific expression of IRG proteins in SkMCs and fibroblasts. The results show that both SkMCs and fibroblasts significantly upregulated Irga6 following stimulation with IFN-γ (Fig. 6A). Irga6 mRNA levels were particularly high in C2C12 SkMCs after IFN-γ stimulation whereas similar levels were detected in pSMC and fibroblasts. Likewise, Irgm3 (i.e. IGTP) was strongly upregulated in SkMCs following treatment with IFN-γ (data not shown). Confocal microscopy of fluorescently labeled Irga6 confirmed expression of the protein within 2 to 4 hours of stimulating C2C12 SkMCs with IFN-γ, with levels further increasing thereafter (Fig. 6B). Importantly, starting at 8 hours after stimulation, Irga6 accumulated at vacuoles containing *Toxoplasma* parasites of the mouse-avirulent NTE strain, and protein levels as well as the percentages of positive vacuoles further increased thereafter (Fig. 6B,C). In comparison, the percentage of Irga6-positive vacuoles was much lower in IFN-γ-stimulated fibroblasts (Fig. 6C), or was almost not detected in non-stimulated cells (Fig. 6B,C). Since IRG proteins have been shown to preferentially accumulate at PVs containing avirulent, but not virulent *T. gondii* strains [38], [39], [40], we also examined IRG protein deposition on PVMs surrounding β-galactosidase-expressing transgenic RH parasites in SkMCs. Within two hours of infection, Irga6 localized to a small but significant number of RHβGal-containing vacuoles in IFN-γ-activated C2C12 SkMCs (Fig. S1). The number of Irga6-positive PVs subsequently increased, and percentages of positive PVs harboring RHβGal parasites reached even higher levels than those containing NTE parasites (Fig. S1). As expected, IRG protein deposition on RHβGal vacuoles was absent in non-activated SkMCs, and this correlated with unrestricted parasite replication (Fig. S1A). Following loading of PVs containing avirulent NTE parasites with IRG proteins in IFN-γ-activated SkMCs, we regularly recognized vacuoles that appeared to be disrupted (Fig. 6D, arrow). Such vacuoles were characterized by Irga6 being located in aggregate-like structures that did not completely surround the parasite as described previously in IFN-γ-treated astrocytes [25]. Furthermore, punctate structures that clearly reacted with anti-*Toxoplasma* serum became evident suggesting disintegration of the parasite (Fig. 6D, small arrow heads).

**Figure 7:**
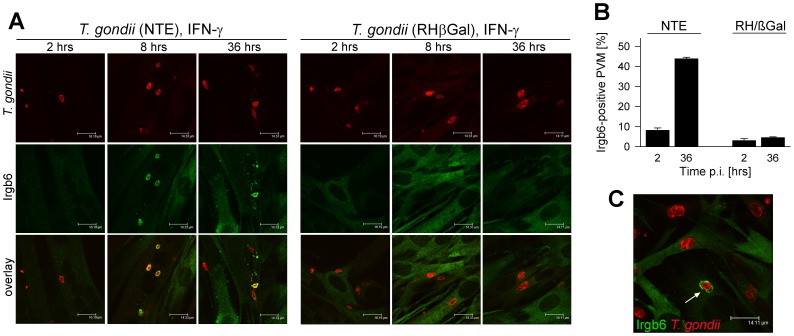
Differential targeting of GTPase Irgb6 to parasitophorous vacuoles of type I and type II *Toxoplasma* strains in SkMCs. Differentiated C2C12 SkMCs were infected with *T. gondii* RHβGal or NTE parasites and were activated with 100 U/ml IFN-γ at the time of infection. After fixation at different time points p.i., Irgb6 (green fluorescence) and *T. gondii* (red fluorescence) were immunolabeled and analysed by confocal laser scanning microscopy. (A) Representative images of PVs of NTE and RHβGal parasites from one out of two independent experiments are depicted. (B) Percentages of Irgb6-positive parasitophorous vacuolar membranes (PVM) were determined microscopically by examining at least 100 vacuoles per sample; data represents means ± S.E.M. (n = 2). (C) Irgb6-positive RHβGal vacuoles could be rarely observed; a RHβGal vacuole showing early signs of disruption is indicated.

Despite the loading of distinct IRGs on PVs harboring type I, i.e. virulent *T. gondii*, they nevertheless avoid vacuole disruption [37], [39], at least in part by phosphorylating IRGs through the parasite kinase ROP18 [41], [42]. We therefore next compared loading of Irgb6 on vacuoles containing NTE and RHβGal *T. gondii*, since its presence at PVMs correlates with subsequent PV disruption and parasite death [37], [39], [40]. The results show that Irgb6 was efficiently deposited on vacuoles containing NTE but not RHβGal parasites (Fig. 7A). Loading of PVs with Irgb6 was already detected at 2 hours after infection/activation with IFN-γ and the percentage of Irgb6-positive PVs rose thereafter in NTE-infected SkMCs to approximately 45% (Fig. 7B). In contrast, in RHβGal-infected SKMCs, the percentage of Irgb6-positive vacuoles did not exceed 5%. We nevertheless observed a small proportion of SkMCs with RHβGal-containing PVs that also accumulated Irgb6 and that showed signs of PV disruption, e.g. rough or even disrupted PVM labeling (Fig. 7C) [25]. Together, the data strongly suggested that IRG-mediated disruption of parasite-containing vacuoles may contribute to the IFN-γ-mediated defense of SkMCs primarily but not exclusively against avirulent *T. gondii*.

**Figure 8:**
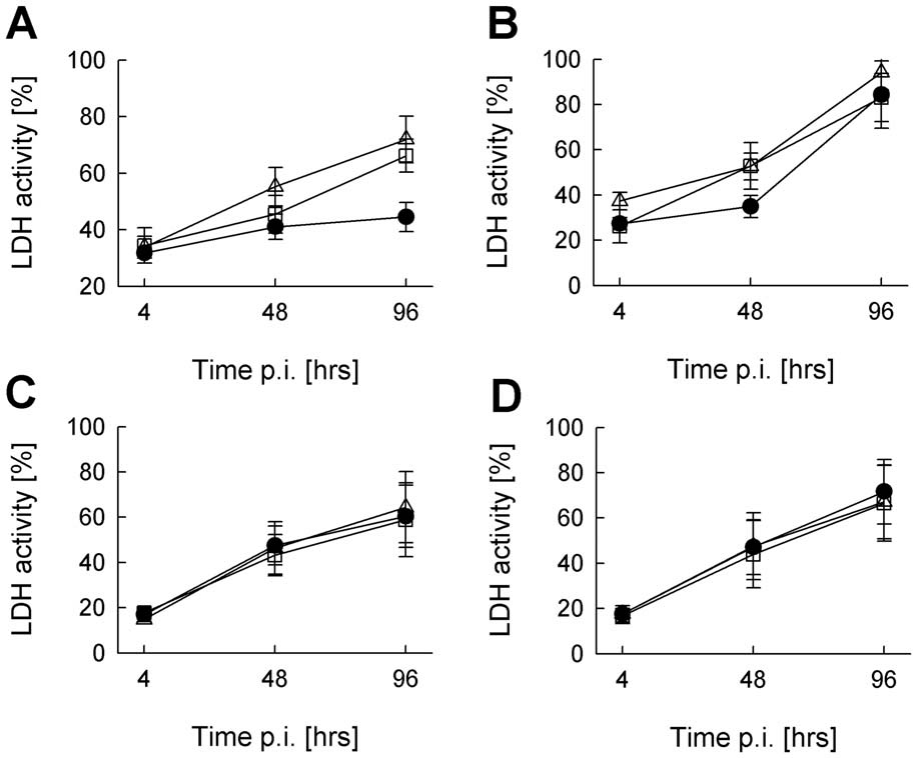
IFN-γ does not accelerate necrotic cell death in *Toxoplasma*-infected SkMCs. Differentiated C2C12 SkMCs (A,B) and NIH/3T3 fibroblasts (C,D) were either infected with *T. gondii* (NTE strain, parasite-host cell ratio 2∶1; B,D), or were mock-infected (A,C). At the time of infection, cells were stimulated with IFN-γ alone (open squares) or combined with TNF (open triangles), or were left non-stimulated (closed circles). Lactate dehydrogenase (LDH) activity was monitored in culture supernatants at different time points of infection. Maximal LDH activity of each sample was determined in parallel by complete cell lysis. LDH activities in the supernatants of experimental samples are expressed as percentages of the maximal activity; data represents means ± S.E.M. from three independent experiments.

Lysis of *Toxoplasma* vacuoles by IRG activity and parasite death can subsequently lead to necrotic death of the host cell [37]. Therefore, we next examined whether IFN-γ alone or in combination with TNF induces cell death in SkMCs or fibroblasts. IFN-γ or IFN-γ plus TNF indeed increased LDH activity in the supernatants of non-infected SkMCs until 4 days of stimulation as compared to untreated controls thus indicating a loss of cell viability (Fig. 8A). More importantly, however, IFN-γ did not specifically decrease the cell viability in *Toxoplasma*-infected SkMCs, as it would have been expected if IRG-mediated disruption of parasite PVs led to host cell death (Fig. 8B). Instead, infection of C2C12 cells with *T. gondii* led to an increase of LDH activity particularly at later time points of infection, and this occurred in both cells that had been activated with IFN-γ or not. LDH activity also increased time-dependently in control fibroblasts irrespective of whether cells had been infected or not with *T. gondii* and had been treated or not with IFN-γ or IFN-γ along with TNF (Fig. 8C,D). Thus, host cell viability was not compromised as a result of IRG activity and disruption of parasite-containing PVs in IFN-γ-activated SkMCs.

## Discussion

Skeletal muscle is one of the preferred tissues that sustains the development and long-term survival of *Toxoplasma* tissue cysts [7]. Here, we have explored the cell-autonomous immune responses of primary and permanent mouse-derived SkMCs after infection with *T. gondii*. The results for the first time establish the immunocompetence of mature muscle cells during infections with both avirulent and virulent *T. gondii* strains. IFN-γ, but not TNF is pivotal in activating innate defense mechanisms in SkMCs and in restricting parasite propagation. We provide evidence that NO production by iNOS and loading and subsequent disruption of *Toxoplasma* containing PVs by IRGs (i.e. p47 GTPases) fulfil anti-parasitic activities in SkMCs after stimulation with IFN-γ. Remarkably, inhibition of parasite replication in SkMCs after stimulation with IFN-γ does not accelerate bradyzoite formation. These results strongly suggest that during toxoplasmosis, SkMCs are active players rather than passive targets of the local immune response towards *Toxoplasma* thereby determining the host-parasite interaction in muscle tissue.

IFN-γ-activated, mononuclear phagocytes are the major mediators of resistance to *T. gondii* [43]. However, IFN-γ-responsiveness of non-hematopoietic cells is also required for mice to survive a *Toxoplasma* infection [20]. In this study, we prove that SkMCs are capable to considerably inhibit propagation of *T. gondii* after stimulation with IFN-γ. They may thus actively contribute to the local host defense in muscle tissue. While this is rather unlikely to be critical for host survival, immune responses to *T. gondii* in skeletal muscle may however significantly impact the ability of the parasite to persist within this tissue. Although species-specific differences in effector molecules of the host response clearly exist (see below), we nevertheless assume that our findings in general also apply for the immune responses in SkMCs from animals used for human consumption such as pigs, sheep, goats, poultry and others. Anti-parasitic cell-autonomous immunity in SkMCs from livestock may thus have major implications for transmission of *T. gondii* to humans via the ingestion of undercooked or cured meat products. In addition, it also establishes SkMCs as active participants during inflammatory myopathies, e.g. polymyositis or dermatomyositis, during toxoplasmosis as described in humans and animals [10], [11], [12], [13], [14].

The capability of SkMCs to restrict parasite propagation after activation with IFN-γ is in sharp contrast to neurons which is another preferred cell type for *Toxoplasma* long-term persistence [44], [45], and which failed to inhibit the parasites' replication after activation with IFN-γ and/or TNF [46]. This indicates that responsiveness of the infected host cell to IFN-γ and/or TNF appears not to be of major relevance for the preferential formation of tissue cysts and long-term persistence of *T. gondii* within brain and muscle tissues. This view is further supported by the finding that treatment of SkMCs with IFN-γ alone or combined with TNF even diminished the conversion from tachyzoites to potentially persisting bradyzoites. The latter contrasts to the triggering of bradyzoite formation in mouse macrophages after stimulation with IFN-γ [33]. In distinct cell types including SkMCs and neurons, however, cell intrinsic factors may instead favor stage differentiation and persistence of *Toxoplasma* tissue cysts as also suggested earlier [2], [47]. Recently, it has been indeed shown that upregulation of the cell division autoantigen-1 following treatment of human fibroblasts with a trisubstituted pyrrole, triggers stage differentiation and *Toxoplasma* cyst formation [48]. It should be mentioned that from the present study we cannot completely rule out the possibility that long-term stabilization of tissue cysts within SkMCs is achieved by activation with IFN-γ.

Our data shows that IFN-γ exerts its effect primarily on the parasite replication in SkMCs but not the parasite invasion into SkMCs since initial infection rates were similar irrespective of IFN-γ stimulation. Only at later time points of infection, IFN-γ-dependent differences in the proportion of infected SkMCs became apparent which were most likely due to the reduction in parasite replication after IFN-γ activation, thereby avoiding host cell lysis and subsequent invasion of new host cells. *Toxoplasma* gains access into its host cell by an active parasite-driven process and we confirm that this remains unaffected by stimulation of the host cell with IFN-γ or not. Instead, IFN-γ-induced iNOS expression and activity contributed to the toxoplasmacidal effect in SkMCs as revealed by pharmacological inhibition. Reactive nitrogen intermediates including NO exert important toxoplasmacidal activities against both type I (mouse-virulent) and type II (mouse-avirulent) parasites in mice *in vitro* and *in vivo* [23], [24], [35], [36], [49], and our results suggest that this holds also true for the local immune response in muscle tissue. Stimulation of SkMCs with IFN-γ led to much higher levels of iNOS transcripts and NO as compared to fibroblasts. Consequently, inhibition of iNOS partially abrogated the effect of IFN-γ on parasite replication in SkMCs but not in fibroblasts. Inflammatory cytokines including IFN-γ also triggered iNOS activity in cardiomyocytes from mice and rats and exerted microbicidal activity against a flagellate parasite, *Trypanosoma cruzi* [50], [51]. Thus, iNOS and NO appear to represent a general defense mechanism of muscle cells against intracellular pathogens at least in mice. Since NO production also modulates the expression of chemokines in muscle cells [52] it will be interesting to see whether iNOS activity may also be involved in the pathogenesis of inflammatory myopathies during toxoplasmosis.

Another mechanism that we describe exerting anti-*Toxoplasma* activity in SkMCs is the loading of parasitophorous vacuoles with IFN-γ-dependent IRG proteins and subsequent disruption of vacuoles. This protein family plays a pivotal role in the immune defense against *Toxoplasma* and other intracellular pathogens. Importantly, several reports indicate that the IRG protein family only confers resistance against mouse-avirulent *T. gondii* strains whereas virulent *T. gondii* evade IRG-mediated destruction [37], [38], [39], [40], at least partially via phosphorylation by the rhoptry kinase ROP18 [41], [42]. Hence, mice deficient in individual members of the IRGs rapidly succumb during acute infection with an avirulent *T. gondii* strain [53], [54], [55]. IRGs can be induced in a variety of tissues [53] and expression in both hematopoietic and non-hematopoietic cells is required for resistance against *Toxoplasma* [56]. Here, we show for the first time that Irga6 and Irgm3 were strongly induced in SkMCs following treatment with IFN-γ. More importantly, both Irga6 and Irgb6 rapidly accumulated on the membrane of vacuoles containing avirulent NTE parasites and together with other IRGs may subsequently mediate disruption of the parasitophorous vacuole as also suggested herein. Vacuoles containing virulent RHβGal, on the other hand, efficiently accumulated only Irga6 but not Irgb6, and signs of vacuole disruption were also only rarely observed. Coating of PVs with IRGs in a consistent hierarchy is necessary and supposedly also sufficient [38] to mediate vacuole disruption and parasite death. Loading of PVs with Irgb6 is an early marker of subsequent vacuole disruption [37], [38], [39], largely discriminates between vacuoles containing virulent type I or avirulent type II and III *T. gondii* [37], [38], [39], and is necessary for efficient defense of primed macrophages against *Toxoplasma* [42]. Our data is thus consistent with the view that the IRG system is a critical strain-dependent defense mechanism of mice against *T. gondii*. In our study, we extend these previous findings by demonstrating that it also operates in IFN-γ-activated SkMCs infected with type II and presumably also type III parasites. We indeed provide circumstantial evidence for disrupted *Toxoplasma* vacuoles in IFN-γ-activated SkMCs infected with NTE parasites and we propose that this contributes to the host defense against *T. gondii* in muscle tissue. It should be mentioned that in activated SkMCs, we occasionally also observed Irgb6-positive vacuoles harboring virulent RHβGal parasites and that we also recognized signs of disrupting RHβGal PVs. Thus, evasion of IRG-mediated destruction is not an absolute trait of virulent *T. gondii* parasites as also reported by others [37], [38]. Although present throughout the vertebrates, the IRG protein family has been lost or largely reduced in birds and humans, respectively [55]. It remains to be determined whether in these species the loss of a functional IRG system is compensated by another family of GTPases, i.e. the p65 guanylate-binding proteins (GBPs) [57]. In addition, a possible contribution of GBPs in the local immune response to *Toxoplasma* in SkMCs from IRG-proficient animals, e.g. mice [27], deserves closer attention.

The parasite and host cell fate after IRG-mediated disruption of the PV is a matter of debate and far from having been solved [55]. In IFN-γ-primed mouse astrocytes, parasites released from disrupted PVs directly degenerate in the host cell cytosol [25], [58] or – within a certain time frame post infection – can egress from the host cell [58]. After IRG-mediated PV disruption in activated fibroblasts, degenerating parasites in the host cell cytosol have been shown to induce a necrotic death of the host cell [37]. In IFN-γ-activated SkMCs, we did not recognize any signs of parasite egress or a decrease of host cell viability that could be attributed to IRG activity. This can be due to the fact that we activated the SkMCs at the time point of infection instead of using IFN-γ-primed cells for infection as in the studies described above [37], [58]. In addition, there is clear evidence for cell type-specific differences in the mechanisms of IRG-mediated resistance [55]. Thus, the exact modes of IRG-mediated host resistance in SkMCs remains to be determined.

In conclusion, SkMCs have to be considered potent effector cells of the local innate immune response to *T. gondii*. After stimulation with IFN-γ, they considerably restrict parasite replication by inducing iNOS- and IRG-mediated activities without accelerating bradyzoite formation. Thus, IFN-γ-regulated cell-autonomous immunity of SkMCs rather limits parasite abundance than facilitating tissue cyst formation during *Toxoplasma* colonization of muscle tissue. Differences in the SkMCs immune response to the parasite may also determine the significance of different host species for transmission of *Toxoplasma* to humans via undercooked or cured meat products.

## Materials and Methods

### Ethics statement

All animal work has been conducted according to relevant national and international guidelines and was approved by the Niedersächsisches Landesamt für Verbraucherschutz und Lebensmittelsicherheit (Project 509.42502/01-T-23.05).

### Isolation of primary SkMC and differentiation of C2C12 cells

Primary SkMC were isolated from NMRI mouse embryos 18–19 days after conception as described previously [2]. Briefly, thigh muscles were minced in Ringer solution and digested with 0.05% trypsin, 0.01% EDTA in PBS, pH 7.2. After addition of DMEM, 10% heat-inactivated fetal calf serum (FCS) and centrifugation, cells were incubated for 45 min in DMEM supplemented with 9% horse serum, 5% FCS, 2.5 mM CaCl_2_, 100 U/ml penicillin and 100 µg/ml streptomycin in tissue culture flasks. Non-adherent cells enriched for myoblasts were then seeded at 1.5×10^4^ per well in 96-well flat bottom tissue culture plates, at 3×10^4^ per well in 24-well plates containing 13-mm glass cover slips or at 1.5×10^5^ per well in 6-well plates. In order to facilitate adherence of SkMC, glass cover slips were pre-coated using 0.5 mg/ml poly-L-ornithine hydrobromide in 150 mM boric acid/NaOH, pH 8.3 and 15 µg/ml mouse laminin. Unless stated otherwise, cells were cultured for 6 days in order to enable the formation of myotubes.

C2C12 myoblasts (European Collection of Animal Cell Cultures (ECACC), Salisbury, UK) were propagated in DMEM supplemented with 10% FCS, 100 U/ml penicillin and 100 µg/ml streptomycin. Cells were seeded at 4×10^3^ per well in 96-well tissue culture plates, at 2.5×10^4^ per well in 24-well plates containing 13-mm glass cover slips or at 1×10^5^ per well in 6-well plates. After 24 hours, culture medium was exchanged to DMEM, 2% horse serum and antibiotics as above and cells were allowed to differentiate to myotubes during the next 120 hours. NIH/3T3 mouse fibroblasts (ECACC) were used as control cells and were cultivated in the same medium as the SkMCs.

### Parasite infections and activation of host cells

Parasites of the mouse-avirulent type II strain NTE or the mouse-virulent type I strain RH/*pSAG-lacZ* expressing β-galactosidase under control of the SAG1 promoter [59] were co-cultured with L929 mouse fibroblasts as host cells in RPMI 1640 supplemented with 1% FCS and antibiotics (as above). Before infection of SkMCs or control cells, parasites were separated from host cells by differential centrifugation as described previously [60]. Host cells were infected at a parasite-to-host cell ratio of 2∶1. At the time of infection, they were activated with 0.1 to 100 U/ml mouse IFN-γ (R&D Systems, Wiesbaden, Germany) alone or in combination with 100 pg/ml TNF (BD Biosciences, Heidelberg, Germany), or were left non-stimulated. Endogenous NO production was in some experiments inhibited by treating cells with 100 µM l-N^6^-(1-iminoethyl)-lysine (l-NIL; Sigma-Aldrich, Munich, Germany) starting at 1 hour prior to infection.

### Quantification of lacZ transgenic parasites

Parasite growth of β-galactosidase-expressing *T. gondii* was quantitated by a colorimetric chlorophenol red-β-d-galactopyranoside (CPRG) assay. To this end, cells were fixed by adding an equal volume of 4% formaldehyde, 250 mM NaCl in Hanks Balanced Salt Solution (HBSS), pH 7.6 to the culture medium. After 20 minutes, cells were washed once in HBSS for 15 min and were then incubated in 0.1% Triton X-100, 0.1 mM CPRG, 5 mM MgCl_2_ in HBSS at 37°C. Absorbance was read in a microplate reader at 560 nm.

### Quantitative RT-PCR

The expression of markers for myogenic differentiation and of putative anti-parasitic effector molecules by SkMCs and control fibroblasts was analyzed by quantitative real-time RT-PCR. To this end, total RNA was isolated from *Toxoplasma*-infected and non-infected SkMCs or NIH/3T3 control cells using the GenElute Mammalian Total RNA Isolation kit as recommended (Sigma-Aldrich, Munich, Germany). Following reverse transcription of mRNA using the Omniscript RT kit (Qiagen, Hilden, Germany) and oligo(dT) primers, serial dilutions of cDNA were amplified by real-time PCR (LightCycler, Roche Diagnostics, Mannheim, Germany) using the SYBR Green FastStart DNA Master^Plus^ kit. Mouse-specific primer pairs used in this study are specified in Table 1. The relative fold change of transcript levels following IFN-γ stimulation was calculated according to the formula Ratio  =  (E_target_)^ΔCPtarget(control-stimulated)^/(E_ref_)^ΔCPref(control-stimulated)^, where the reference was β-actin and the target iNOS, Irga6 or Irgm3 (i.e. IGTP) [61]. Likewise, the level of Myf5, MyoD or myogenin transcripts during myogenic differentiation was compared to that before differentiation and was normalized to β-actin mRNA using the same formula as above.

**Table 1:** Primers used in this study for qPCR.

Gene	NCBI accession no.	Forward primer	Reverse primer
*MYOGENIN*	NM_031189	AGCGGCTGCCTAAAGTGGA	CAGCCGCGAGCAAATGAT
*MYOD1*	NM_010866	GCGCCGCTGCCTTCTACG	CTCGACACAGCCGCACTCTTC
*NOS2*	NM_010927	CGAGGAGCAGGTGGAAGACTATTT	GAGAGGGGAGGGAGGAGAGGA
*IIGP1* (syn. *IRGA6*)	NM_021792	TTCCCTAGCTGGCAATCGTCAA	GGCTTTATGTGGGTTCTGGGTATC
*IGTP* (syn. *IRGM3*)	NM_018738	TGAACAAGTTCCTCAGGGTTC	TGAAGGAACGGGCTATTACAG
*ACTB*	NM_007393	GATGACCCAGATCATGTTTGAGAC	TGCTCGAAGTCTAGAGCAACATAG

### SDS-PAGE and immunoblotting

In order to analyze the differentiation of SkMCs and the intracellular development of *T. gondii*, parasite-infected or non-infected SkMCs and fibroblasts were isolated by EDTA/trypsin treatment and equal amounts of cells were lysed for 1 hour in 1% Triton X-100, 0.15 M NaCl, 50 mM Tris/HCl, pH 8.0, 50 mM NaF, 5 mM sodium pyrophosphate, 1 mM PMSF, 1 mM EDTA, 1 mM sodium orthovanadate, and 5 µg/ml each of leupeptin, aprotinin and pepstatin. After centrifugation, soluble proteins were separated by standard SDS-PAGE and transferred to nitrocellulose by semidry blotting. Non-specific binding sites were blocked for 2 hours with 5% dry skimmed milk, 0.2% Tween-20, 0.02% NaN_3_ in PBS, pH 7.4. Membranes were incubated overnight at 4°C with 1.5 µg/ml mouse monoclonal anti-MyoD, 1.5 µg/ml mouse monoclonal anti-myogenin (both from BD Biosciences, Heidelberg, Germany), rabbit polyclonal anti-myosin heavy chain (H-300, 1∶1.000; Santa Cruz Biotechnology, Heidelberg, Germany), mouse monoclonal anti-actin (clone C4; kindly provided by J. Lessard, Cincinatti, OH), rabbit polyclonal anti-*T. gondii* (1∶10.000) or rabbit polyclonal anti-SAG1 (1∶1.000) diluted in 5% dry skimmed milk, 0.05% Tween-20 in PBS, pH 7.4. After washing, primary antibodies were detected with horseradish peroxidase-conjugated anti-mouse or anti-rabbit IgG (Dianova, Hamburg, Germany). Thereafter, enhanced chemiluminescence reagent (GE Healthcare, Freiburg, Germany) was added and digital images obtained using a LAS-4000 luminescent image analyzer (Fujifilm, Düsseldorf, Germany).

### Immunofluorescence staining and confocal microscopy

The intracellular development of *T. gondii* as well as the expression of host cell proteins was determined by immunofluorescence microscopy. Infected SkMCs and fibroblasts or non-infected controls were fixed with 4% paraformaldehyde in 0.1 M sodium cacodylate, pH 7.4 for 1 hour and quenched for 10 minutes in 50 mM NH_4_Cl in PBS, pH 7.4. Thereafter, cells were permeabilized and unspecific binding sites blocked for 1 hour using 0.1 mg/ml saponin, 1% BSA in PBS. Cells were then incubated for 1 hour with 5 µg/ml anti-MyoD, 5 µg/ml anti-myogenin or 5 µg/ml anti-desmin mouse monoclonal antibodies (all from BD Biosciences), rabbit polyclonal anti-myosin heavy chain (1∶100; Santa Cruz Biotechnology), or with rabbit polyclonal anti-*T. gondii* (1∶1.000) alone or combined with rat monoclonal CC2 recognizing bradyzoite-containing but not tachyzoite-containing PVs [62], or with mouse polyclonal anti-*T. gondii* (1∶100) combined with rabbit polyclonal anti-Irga6 (1∶8.000) or anti-Irgb6 (1∶4000; anti-IRGs kindly provided by J. Howard, Cologne, Germany) diluted in 1% BSA, 0.1 mg/ml saponin in PBS. The cells were washed and were then incubated for 1 hour with Cy2-conjugated or Cy3-conjugated donkey F(ab')_2_ fragment anti-rabbit IgG or anti-mouse IgG (Dianova, Hamburg, Germany). In some experiments, the total cell population was labeled with 5 µg/ml propidium iodide in PBS. The cells were mounted with Mowiol 4-88 (Calbiochem-Novabiochem, Bad Soden, Germany) and were then examined by confocal laser scanning microscopy using Leica TCS SP2.

### Griess reaction

Nitrite as a reliable marker for NO production was measured by the Griess assay as described previously [24]. Briefly, 100 µl of cell culture supernatants from *T. gondii*-infected or non-infected SkMCs or NaNO_2_ standards (1–128 µM) were incubated in duplicate with an equal volume of 0.5% sulfanilamide, 0.05% naphthyldiamine dihydrochloride in 5% H_3_PO_4_. The absorbance was read at 560 nm after 5 minutes in a microplate reader.

### Cytotoxicity assay

The integrity of *T. gondii*-infected or non-infected SkMCs and control fibroblasts after stimulation with IFN-γ was determined using a colorimetric Cytotoxicity Detection Kit^Plus^ as recommended by the manufacturer (Roche Diagnostics, Mannheim, Germany). Briefly, the activity of lactate dehydrogenase (LDH) in cell culture supernatants was measured in triplicate by co-incubation for 30 min with an equal volume of reaction mixture, consisting of a diaphorase/NAD^+^ mixture in the assay dye solution. Optical density was determined at 490 nm. The LDH activity in supernatants from experimental samples was expressed as the percentage of the maximal activity which was determined by disrupting the cells in lysis solution (Roche) prior to measuring LDH activity.

### Statistical analysis

Results are expressed as means ± S.E.M. of at least three independent experiments unless otherwise indicated. Significant differences between mean values were identified by the Student's *t*-test. *P* values of less than 0.05 were considered significant.

## Supporting Information

Figure S1 **Immunity-related GTPase Irga6 target parasitophorous vacuoles containing transgenic RHβGal parasites in IFN-γ-activated SkMCs.** Differentiated C2C12 SkMCs were infected with *T. gondii* RHβGal or NTE parasites at a parasite-host cell ratio of 2∶1 as indicated and were concomitantly activated with 100 U/ml IFN-γ or were left non-activated. C2C12 cells were fixed at different time points after infection, and Irga6 (green fluorescence) and *T. gondii* (red fluorescence) were immunolabeled. (A) Representative images of RHβGal-infected SkMCs were recorded by confocal laser scanning microscopy. (B) Percentages of Irga6-positive parasitophorous vacuolar membranes (PVM) were determined microscopically by examining at least 100 vacuoles per sample in C2C12 SkMCs infected with either NTE or RHβGal parasites. Data represents means ± S.E.M. from two independent experiments.
(TIF)
