# Supplementary figures and images for: Interferon-γ Restricts Toxoplasma gondii Development in Murine Skeletal Muscle Cells via Nitric Oxide Production and Immunity-Related GTPases
Source: PLoS One. 2012 Sep 14;7(9):e45440. doi: 10.1371/journal.pone.0045440 (PMC3443239; doi:10.1371/journal.pone.0045440)

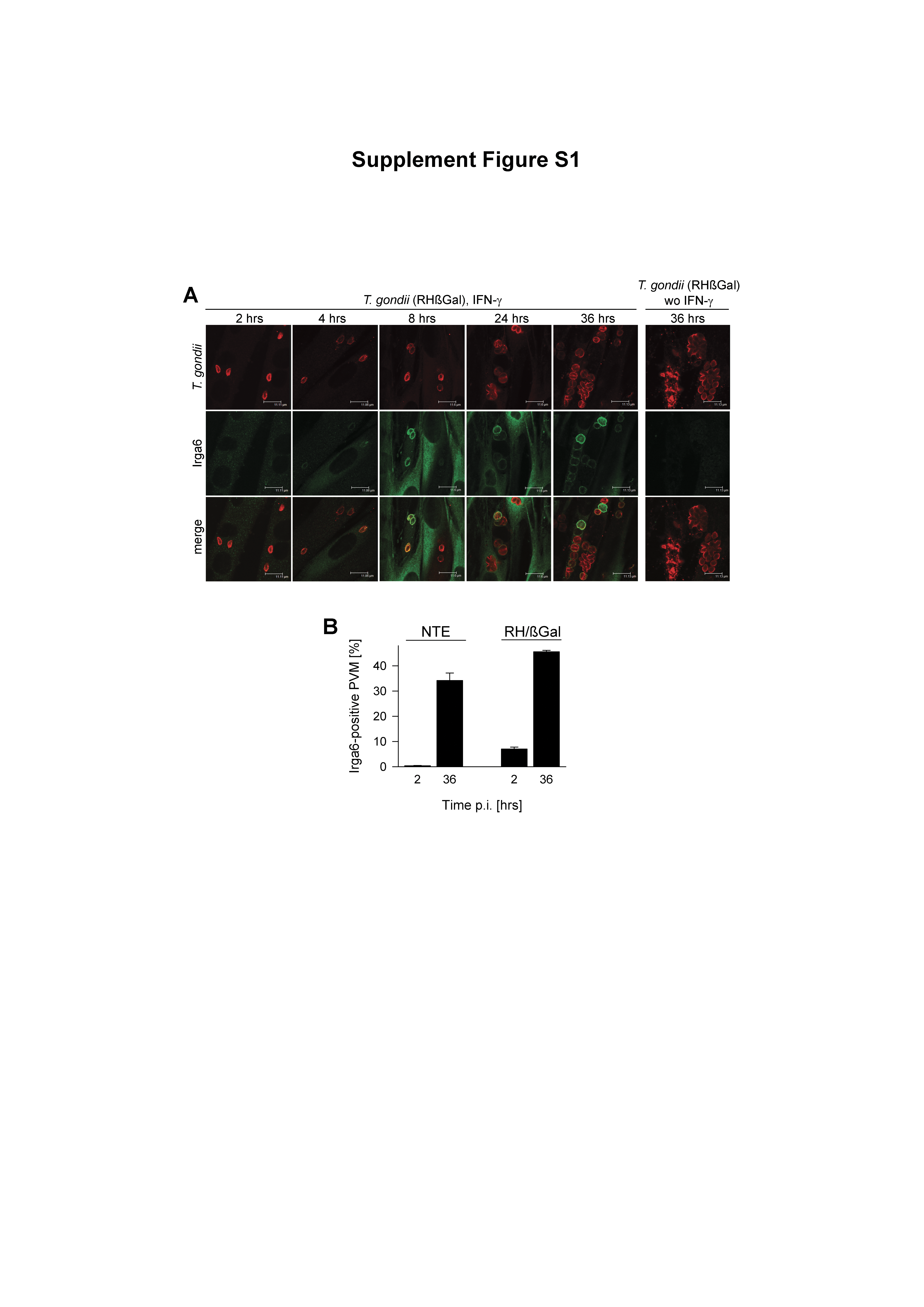

Supplement: Figure S1 — Immunity-related GTPase Irga6 target parasitophorous vacuoles containing transgenic RHβGal parasites in IFN-γ-activated SkMCs. Differentiated C2C12 SkMCs were infected with T. gondii RHβGal or NTE parasites at a parasite-host cell ratio of 2∶1 as indicated and were concomitantly activated with 100 U/ml IFN-γ or were left non-activated. C2C12 cells were fixed at different time points after infection, and Irga6 (green fluorescence) and T. gondii (red fluorescence) were immunolabeled. (A) Representative images of RHβGal-infected SkMCs were recorded by confocal laser scanning microscopy. (B) Percentages of Irga6-positive parasitophorous vacuolar membranes (PVM) were determined microscopically by examining at least 100 vacuoles per sample in C2C12 SkMCs infected with either NTE or RHβGal parasites. Data represents means ± S.E.M. from two independent experiments. (TIF) [file pone.0045440.s001.tif]
